# Metal-based non-enzymatic systems for cholesterol detection: mechanisms, features, and performance

**DOI:** 10.1039/d4ra04104f

**Published:** 2024-08-05

**Authors:** M. Ameen Sha, P. C. Meenu, H. Haspel, Z. Kónya

**Affiliations:** a Department of Applied and Environmental Chemistry, Faculty of Science and Informatics, University of Szeged Rerrich Béla tér 1 Szeged H-6720 Hungary haspel@chem.u-szeged.hu shaalameen@gmail.com mameensha@yahoo.com konya@chem.u-szeged.hu; b Department of Chemistry, Birla Institute of Technology and Science Hyderabad Campus 500078 India meenupc06@gmail.com; c HUN-REN-SZTE Reaction Kinetics and Surface Chemistry Research Group, University of Szeged Rerrich Béla tér 1 Szeged H-6720 Hungary

## Abstract

Metal based catalysts and electrodes are versatile tools known for their redox properties, catalytic efficiency, and stability under various conditions. Despite the absence of significant scientific hurdles, the utilization of these methods in cholesterol detection, particularly in non-enzymatic approaches, has been relatively underexplored. To this end, there is a pressing need to delve deeper into existing metal-based systems used in non-enzymatic cholesterol sensing, with the goal of fostering the development of innovative practical solutions. Various electrode systems, such as those employing Ni, Ti, Cu, Zn, W, Mn, and Fe, have already been reported for non-enzymatic cholesterol detection, some of them elucidated sensing mechanisms and potential in physiological detection. A detailed mechanistic understanding of oxide-based cholesterol sensors, along with the methodologies for constructing such systems, holds promise of advancing the exploration of practical applications. This review aims to provide a broad perspective on metal oxide systems and their characteristics that are conducive to non-enzymatic cholesterol sensing. It is intended to serve as a springboard with offering a guide to the design and development of efficient and sensitive electrochemical cholesterol sensors.

## Abstract 2

Metal based catalysts and electrodes are versatile tools known for their redox properties, catalytic efficiency, and stability under various conditions.

## Graphical abstract

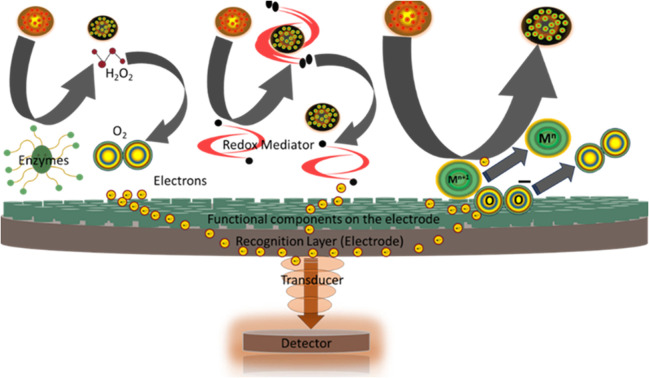

## Introduction

Cholesterol is essential for human life, yet when its levels exceed a critical threshold within the body, it can become a substantial hazard to human well-being. Maintaining a controlled cholesterol intake is vital for supporting crucial physiological functions. Nonetheless, lifestyle choices and dietary habits can contribute to an excessive accumulation of cholesterol in the bloodstream, giving rise to significant health issues. Timely identification of cholesterol levels in the human body, along with appropriate interventions, is of great importance for preserving health and safeguarding human lives. In addition to traditional chemical techniques, enzymatic assay, enzymatic calorimetry, microphotometry, gas chromatography, liquid chromatography, and mass spectrometry are some of the methods used to detect cholesterol.^1–5^ The majority of them require expensive, time-consuming, and complex techniques for the effective and precise detection of cholesterol. At the same time electrochemical sensors are excellent substitutes over traditional analytical methods due to their quick response, high reproducibility, low limit of detection (LOD), and wide concentration range of application.^6,7^

When using an electrochemical sensor, the species on the electrode's surface interact chemically with the analyte, in this case cholesterol, and produces an electrical signal of either current, potential, conductivity, or resistivity. Based on their operating principles, these sensors are known as amperometric, potentiometric, conductometric, or impedimetric sensors, respectively.^8^ A component of the electrochemical biosensor, *i.e.*, the transducer, then transforms the signal into a detectable format.

Many different materials have been investigated for the construction of enzymatic, non-enzymatic, and redox-mediated electrochemical biosensors.^9–16^ Non-enzymatic sensors are receiving considerable attention due to their excellent characteristics, such as the ability to incorporate metals, metal oxides, and composites as electrocatalysts, and strong interaction between the modified electrode and the analyte. This results in high conductivity, biocompatibility, and facile device miniaturization, while these structures are generally unaffected by atmospheric conditions. Conductive polymer composites,^17–19^ carbonaceous materials,^20–23^ metal nanoparticles,^24–29^ metal sulphides,^30–32^ metal oxides,^29–36^ and their composites are typical choices of active material for electrochemical biosensors including cholesterol sensors.^37^

Nanomaterials, as the electroactive component in electrochemical sensors, exhibit diverse mechanisms when employed for cholesterol detection. However, unlike enzymatic sensors, active electrodes are the only essential component in non-enzymatic devices. The integration of nanomaterials into sensor systems offers numerous advantages, like customized electrical and optical characteristics, and enhanced sensitivity due to their high surface area. Polyaniline (PANI) or polypyrrole (PPy) are predominantly reported as polymeric constituents in the literature, such as in CuO–PANI with a fibrous murexide matrix,^14^ PANI/MWCNTs/starch modified carbon paste,^15^ mesoporous PANI nanofiber decorated graphene microflowers,^38^ carboxy-functionalized graphene oxide (fGO) decorated with magnetite (Fe_3_O_4_) nanoparticles on PANI,^39^ Ru–phosphate (Ru–Pi)/PPy modified carbon fiber paper,^40^ Au@NiO decorated PPy composite.^41^ Additionally, alternative polymer-based materials, such as poly(ionic liquid) poly(vinylbutylimidazolium)–cobalt polyoxometalate supported on carbon,^16^ poly(3,4-ethylenedioxythiophene) incorporating taurine,^42^ have also been investigated in this context. Carbon-based materials encompass β-cyclodextrin (β-CD)-functionalized graphene and reduced graphene oxide (rGO),^43^ a graphene oxide-derived molecular imprinted polymer (GO-MIP) serving as the active material,^44^ a network of platinum nanoparticle-functionalized carbon nanotubes (CNTs) assembled through layer-by-layer techniques,^45^ CNTs derived from coconut oil,^46^ multi-walled carbon nanotubes (MWCNTs) that have been functionalized with gold nanoparticles (Au NPs),^47^ and a disposable screen-printed carbon electrode (SPCE) constructed from MWCNTs and β-CD.^48^

Here we review non-enzymatic electrochemical cholesterol sensors constructed of metal compounds and composites and focusing on the mechanistic understanding of the interaction between the electrode materials and the analyte. The goal of this review is to facilitate future advancements in the field by offering valuable insights into the underlying physical–chemical processes, and to aid with the development of innovative and high-performance sensor chips.

## Chemistry of electrochemical cholesterol sensors

An electrochemical biosensor operates by detecting the presence or concentration of a specific biological target (analyte) through interactions with specialized materials in its recognition layer. These interactions generate signals that convey information about the analyte. The recognition layer typically comprises an electrode with functional groups capable of binding to the analyte. For instance, in cholesterol detection the electrode surface may feature enzymes that interact with cholesterol molecules or a substance capable of initiating a redox process upon contact with cholesterol. Redox mediators can also be incorporated to enhance the sensitivity of the biosensor when cholesterol molecules attach to the recognition layer.^49–51^ Subsequently, a transducer transforms the resulting physical–chemical or electrochemical interactions into electrical signals. Finally, a processor processes and converts these signals into a digital output, which can be easily displayed and interpreted.

## Sensing mechanism in different types of cholesterol sensors

The foundation of electrochemical cholesterol sensing involved the integration of enzymes, such as cholesterol oxidase (ChOx) and cholesterol esterase (ChE), within the recognition layer. These enzymes were chosen for their capacity to either bind to or undergo reactions with the target cholesterol molecules as part of the sensing process.

**Scheme 1:**
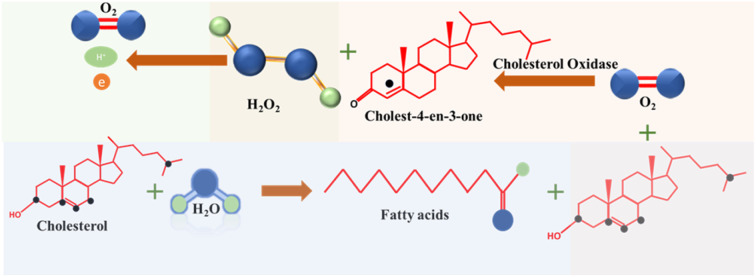
Reactions involved in enzymatic detection of cholesterol.

In enzymatic cholesterol sensors ChE initiates a hydrolysis reaction, breaking down cholesterol ester into free cholesterol and fatty acids, which in turn provides the total cholesterol content in the sample. Simultaneously, a cholesterol molecule undergoes oxidation through the interaction with ChOx, resulting in the production of cholest-4-ene-3-one. Notably, hydrogen peroxide (H_2_O_2_) generated as a byproduct during this redox reaction serves as redox agent for the electrochemical detection of cholesterol at the sensor, eliminating the need for additional redox mediators. *E.g.*, in amperometric sensors current is measured when H_2_O_2_ is oxidized at an applied potential, which in turn correlates with cholesterol concentration. However, in some instances, the current response associated with dioxygen electroreduction may also be used. Chemical reactions taking place in enzymatic cholesterol sensing are depicted in Scheme 1.^8^ A significant anodic potential of approximately +0.7 V *vs.* RHE is required for the oxidation of H_2_O_2_, which represents a significant drawback of this technique. This issue is addressed by incorporating redox mediators alongside the enzymes to facilitate electron transfer.

**Scheme 2:**
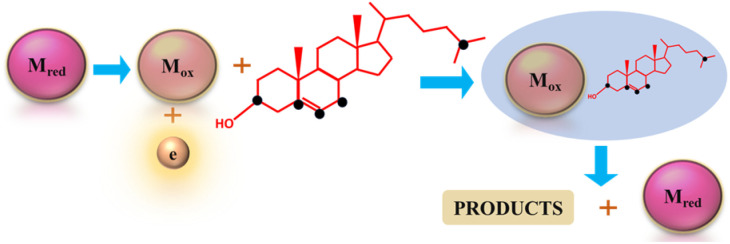
Reactions involved in the redox mediator-based detection of cholesterol.

In the redox mediator based enzymatic detection techniques, ferrocene,^7^ methylene blue,^8^ Prussian blue,^52^ hydroquinone,^53^ poly(*o*-phenylenediamine),^54^ and (hydroxymethyl)ferrocene^55^ were reported to be implemented in the system. Here, the regeneration of the reduced form of the redox mediators by electrical potential is utilized for the detection process. First the oxidized mediator forms an adduct with cholesterol. The latter then gets oxidized into various products, while the redox mediator is electrochemically reduced to generate the signal as shown in Scheme 2.^7^

Horseradish peroxidase (HRP) based bienzymatic mediator-free systems are also capable of sensing cholesterol to some extent.^56–58^ While these enzymatic systems exhibit specificity, sensitivity, and speed in cholesterol sensing, they do face certain limitations. The latter primarily revolves around high costs and limited tolerance to variations in environmental factors, such as pH and temperature. These shortcomings pose challenges when considering the practicality and economic viability of these systems in real-world applications. Consequently, numerous researchers have undertaken efforts to develop simpler, more efficient, and cost-effective non-enzymatic cholesterol sensors.

A non-enzymatic cholesterol sensor comprises a recognition layer devoid of enzymes that interacts with cholesterol molecules. The fundamental role of this layer remains consistent across various materials employed for this purpose, which primarily is to serve as cholesterol oxidants facilitated by electrical processes. These non-enzymatic systems necessitate the use of a conductive electrolyte containing cholesterol to obtain an electrical response during the interaction between the electrode and the analyte. Numerous electrolyte combinations were explored with cholesterol based on their chemical and electrochemical properties. Also, various electrode types, ranging from simple bare electrodes to modified nanostructures, were investigated as recognition layers in non-enzymatic systems.

**Fig. 1:**
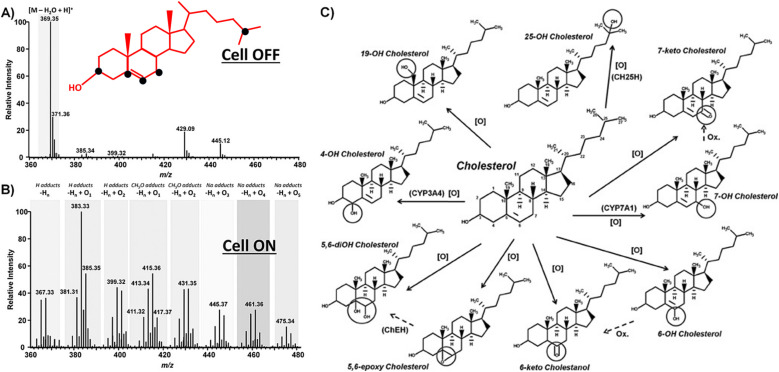
Oxidizable sites (black dots) in the cholesterol molecule along with the MS spectra of cholesterol (100 mmol L^−1^) before (A) and after EC-oxidation (B). The corresponding potential products of cholesterol electrooxidation. (C) Reprinted with permission from ref. 59. Copyright 2016 John Wiley and Sons and Copyright Clearance Center (LN: 5833140916083).

The resulting products and the pathways of electro-oxidation can vary depending on the conditions and materials, since cholesterol contains multiple sites for oxidation. The sites susceptible to oxidation, as depicted in Fig. 1, lead to a diverse array of products during electro-oxidation.^59^ However, the chemistry of all the products has not been completely confirmed, yet.

## Metal-based systems for non-enzymatic detection of cholesterol

Cholesterol sensing can be achieved using a bare Pt or C electrode in combination with an organic medium.^60–63^ However, the resulting product and cholesterol oxidation efficiency vary due to differences in electrode characteristics and the composition of the medium.^60–75^Table 1 overviews non-enzymatic cholesterol oxidation and detection efficiencies (LOD, LOQ), product distributions at the indicated potentials using metal-based electrodes with various supporting electrolytes. Certain data points are not available (NA), as some studies focus on electro-oxidation mechanisms, while others address the direct sensing characteristics. It is also worth noting, that although according to the IUPAC definition, the LOQ/LOD ratio must be 10/3 ≈ 3.33, this relationship is not fulfilled for some of the reported performance metrics in Table 1. These data are marked by asterisks.

**Table 1:** Details of non-enzymatic cholesterol detectors using bare metal or modified electrodes^a^

Electrode	Supporting electrolytes (media)	Cholesterol oxidation products	Oxidation potential (V)	Sensitivity	LOD	LOQ	Ref.
Pt	Glacial acetic acid, sodium perchlorate and sodium acetate	7α-Acetoxycholesterol and 7β-acetoxycholesterol	1.7 (*vs.* SCE)	NA	NA	NA	60
Pt	TBATFB in dichloromethane and cholesterol in glacial acetic acid/acetonitrile	Cholesterol dichlorides/chlorohydrine/dicholesteryl ether/cholesterol acetate/3β-acetylamino derivative of cholesterol	1.75 (*vs.* Ag/0.1 M AgNO_3_)	NA	NA	NA	61
Pencil Pb	Acetonitrile, acetone and lithium perchlorate	Cholesta-4,6-dien-3-one	1.45 (*vs.* SCE)	1455.22 μA mM^−1^ cm^−2^	0.625 mM*	9.375 mM*	64
Au/Pt	Triton X-100, 2-propanol and PBS	NA	0.3–0.2 (*vs.* Ag/AgCl)	226.2 μA mM^−1^ cm^−2^	0.015 mM*	5 mM*	65
Cu_2_S/Cu	Acetate buffers containing cholesterol	NA	NA	62.5 μA mM^−1^ cm^−2^	0.1 μM*	6.8 mM*	66
NiO/graphene	PBS containing isopropanol and Triton X-100	5-Cholesten-3-one	0.55 (*vs.* Ag/AgCl)	40.6 μA mM^−1^ cm^−2^	0.13 μM	40 μM	67
		4-Cholesten-3-one					
(MnO_2_/GR/PGE)	PBS	Cholestenone	−0.24 (*vs.* SCE)	63 869 μA mM^−1^ cm^−2^	0.42 nM	1.2 nM	68
ZnO NRs	PBS	Oxysterols (7β-ketocholesterol, 7α-hydroxycholesterol and 7β-hydroxycholesterol)	+0.5 (*vs.* Ag/AgCl)	4.2 μA mM^−1^ cm^−2^	1.78 mM	9 mM	69
Ag NPs–ZnO NRs				135.5 μA mM^−1^ cm^−2^	0.184 mM		
PMO–NiO/MoS_2_/SPCE	PBS	Oxysterols	−0.27 (*vs.* NA)	7.95 × 10^−6^ A mg^−1^ dL^−1^ cm^−2^	0.24 mg dL^−1^*	15 mg dL^−1^*	70
TiO_2_/GO	PBS	NA	0.619 (*vs.* SCE)	NA	0.05 μM*	0.1 μM*	71
Cu/Ni dispersed carbon nanofiber/polymer nanocomposite	PBS	NA	0.308 (Ag/AgCl (0.3 M KCl))	226.30 μA mM^−1^ cm^2^	2 μg dL^−1^	6.2 μg dL^−1^	72
SiO_2_/Fe_2_O_3_/MWCNT	NA	NA	−0.6 (*vs.* Ag/AgCl)	NA	5 μM	NA	75
Polyindole/WC/SS	NA	NA	0.45 (*vs.* Ag/AgCl)	NA	1.23 × 10^−6^ mol L^−1^	NA	76
Cu_2_O–TiO_2_	PBS and isopropanol	Oxysterols (7*b*-ketocholesterol, 7*a*-hydroxycholesterol and 7*b*-hydroxycholesterol)	−1.04 V (*vs.* Ag/AgCl)	6034.04 μA mM^−1^ cm^−2^	0.05 μM*	622 μM*	77

a * – the IUPAC definition of LOQ/LOD = 10/3 ≈ 3.33 is not fulfilled for these reported values.

## Metal oxide-based non-enzymatic cholesterol sensors – material development, characteristics, and performance

**Fig. 2:**
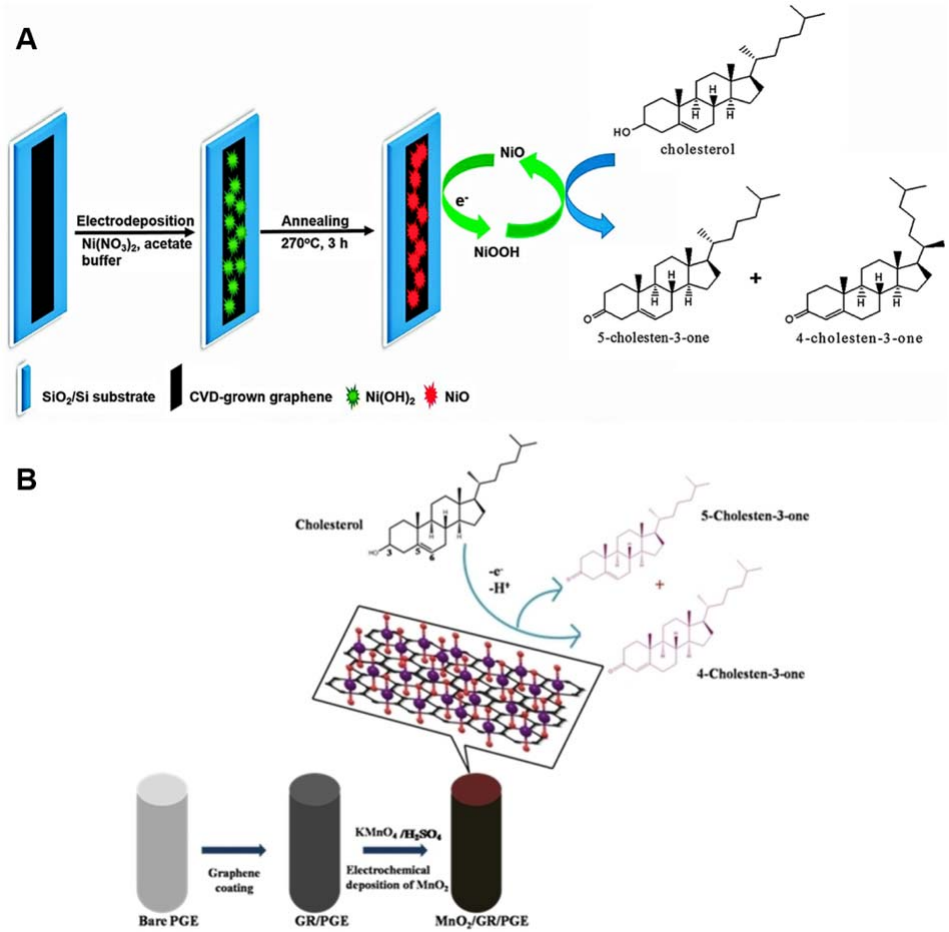
Fabrication of a NiO/graphene composite electrode for cholesterol sensing (A). Reprinted with permission from ref. 67. Copyright 2015 Royal Society of Chemistry (LN: 1507244-1), and the schematics of the electrocatalytic oxidation of cholesterol at MnO_2_/GR/PGE (B), reprinted with permission from ref. 68. Copyright 2020 Wiley-VCH Verlag GmbH & Co. KGaA, Weinheim (LN: 5833150494623).

Rengaraj *et al.* innovatively engineered a flowerlike NiO structure atop high-quality graphene to enable non-enzymatic cholesterol sensing.^67^ Graphene is the optimal support in biosensor applications because of its exceptional carrier mobility, ambipolar behavior, and unique energy structure. The single atom-thick carbon layer was deposited onto copper *via* chemical vapor deposition using methane and oxygen as reagents at ∼1000 °C, and subsequently transferred onto glassy carbon electrode (GCE) by the PMMA method. NiO was then electrodeposited onto the graphene flakes from an electrolyte containing Ni(NO_3_)_2_ with acetate buffering as it is outlined in Fig. 2A. After annealing under air at 270 °C the performance of the developed electrodes was assessed in the absence of any enzymes in a solution of isopropanol and Triton X-100 containing varying concentration of cholesterol in phosphate buffer (PBS) and 1 M KOH by cyclic voltammetry (CV) and chronoamperometry (CA). The fabricated sensor exhibited a detection range spanning from 2 μM to 40 μM cholesterol concentrations with LOD of 0.13 μM and a rapid response time of 5 seconds. The attractive performance can be attributed to the electrocatalytic properties of NiO and the large specific surface area provided by graphene. The reaction mechanism involves the electrochemical conversion of Ni^2+^ to Ni^3+^ by hydroxyl ions in the surrounding medium, which is subsequently followed by the oxidation of cholesterol:^67^

1 NiO + OH^−^ ↔ NiOOH + e^−^

2 Ni^3+^ + cholesterol → Ni^2+^ + cholestenone

This process results in a linear increase in the current associated with cholesterol oxidation as cholesterol concentration rises, and the electrodes exhibit a high sensitivity of 40.6 mA mM^−1^ cm^−2^ with good stability throughout the study.

In the study of Rison *et al.* graphene-modified pencil graphite electrode (PGE) was decorated with MnO_2_ nanoclusters.^68^ Initially, graphene was synthesized through the exfoliation of 60 mesh particle size graphite in acidic medium, followed by heating and exfoliation in a microwave oven. The resulting graphene was further purified using a solvent mixture containing acetone, dimethylsulfoxide, and water. For sensor electrode fabrication, a pre-treated pencil graphite electrode (PGE) was brush-coated with a paste composed of graphene, polyvinylidene chloride (PVDF), and *N*-methyl-2-pyrrolidene (NMP). MnO_2_ was then electrodeposited onto the heat-treated graphene-coated PGE from KMnO_4_/H_2_SO_4_ solution. Electrochemical behavior was investigated at various pHs, and cholesterol sensing performance were assessed over several cycles by performing CV and Differential Pulse Voltammetry (DPV) in PBS buffer and with interfering substances modeling human blood serum. Enhanced electron transfer ability was observed due to the presence of catalytic MnO_2_ as evidenced by the elevated anodic peak current at −0.2 V (*vs.* SCE) when compared to that of the bare electrode. LOD and LOQ were found to be 4.2 × 10^−10^ M and 12 × 10^−10^ M, respectively. Furthermore, the cholesterol sensing process on MnO_2_/G/PGE is diffusion-controlled involving a two-electron transfer mechanism resulting in the oxidation of cholesterol to cholestenone as outlined in Fig. 2B.

ZnO seeds were precipitated directly onto a Pt interdigitated electrode by annealing Zn(CH_3_COO_2_), where then ZnO nanorods were grown from those seeds *via* hydrothermal synthesis. The as-fabricated sensor displayed a cholesterol sensitivity of 4.2 μA mM^−1^ cm^−2^. This was further improved by Ag nanoparticle deposition (AgNPs/ZnO-NR) reaching a sensitivity of 135.5 μA mM^−1^ cm^−2^ along with a remarkably low LOD of 0.184 mM. A reduction in charge transfer resistance (*R*_ct_) was observed in AgNP/ZnO-NRs by EIS, leading to improved sensing performance through the enhanced electron transfer.^69^

Ariyanta *et al.* immobilized a nanocomposite of NiO and MoS_2_ on a screen-printed carbon electrode (SPCE), which was subsequently modified by electrochemically polymerizing poly(methyl orange) (MO) from a MO/PBS solution as a cholesterol-identifying agent. Calibration was done in Triton-X/PBS solutions, and pretreated milk and yogurt samples were electrochemically tested *via* CV and DPV. Negative potential at the n-type semiconductor MoS_2_ increased the electron density in the conduction band, which in turn facilitated electron transfer to the O_2_ molecule converting O_2_ to O_2_^−^. The latter then reacted with H_2_O to produce H_2_O_2_ and hydroxyl radicals, thus initiating cholesterol oxidation to oxysterol. The sensor exhibited a detection range spanning from 25.86 μM to 0.39 mM with LOD and LOQ values of 0.24 mg dL^−1^ and 0.81 mg dL^−1^ at a reasonably high sensitivity of 30.63 μA mM^−1^ cm^−2^, respectively. The obtained cholesterol concentrations were in accordance with those indicated on the packaging, further validating the fabricated sensor electrode.^70^

A gold-decorated nickel oxide (Au@NiO) on PPy nanocomposite was deposited onto glassy carbon electrode (Au@NiO/PPy–GCE) and studied in electrochemical cholesterol sensing *via* CV and DPV. The results revealed a linear detection range spanning from 1.0 × 10^−5^ to 1.0 × 10^−4^ M at a sensitivity of 7.6 μA μM^−1^ cm^−2^ and an impressive LOD of 5.8 × 10^−7^ M. Crucially, interference with various substances such as glucose, dopamine (DA), NaCl, uracil, uric acid, and ascorbic acid did not affect the determination accuracy. This high selectivity was attributed to the combination of exceptional electrocatalytic activity and the vast specific surface area.^41^

A cholesterol sensing electrode was developed specifically for the purpose of investigating a variety of food products, such as vanilla essence, vanilla-flavored biscuits, ice cream, and cake mix. First, TiO_2_ was directly grown onto rGO through a hydrothermal process (rGO–TiO_2_), then polypyrrole was deposited by *in situ* chemical oxidative polymerization. The constructed rGO–TiO_2_/PPy nanocomposite was applied onto GCE and its cholesterol sensing performance was studied by electrochemical measurements, including CV and CA. This electrode exhibited a remarkably low LOD and LOQ of 0.05 μM and 0.1 μM, respectively.^71^

Bairagi and Verma fabricated a poly methyl orange (PMO) dendritic film/Cu/Ni/carbon nanofiber (CNF) electrode for non-enzymatic cholesterol sensing. Cu/Ni bimetal nanoparticles were deposited onto activated carbon fiber (ACF),^73,74^ which was transformed into a Cu/Ni/CNF nanocomposite in a CVD process. The ball-milled composite was cast into a polyvinylacetate (PVAc) metal–carbon–polymer composite film and a dendritic PMO nanofilm was grown through electrochemical polymerization of methyl orange. Here, PVAc served as a binder, and the composite is the active sensing element. The electrode was tested in electrochemical cholesterol sensing in Triton-X/PBS (pH = 7.0) and in human blood samples *via* CV, DPV, and EIS. Impedance spectra showed the diffusion-controlled cholesterol transport toward the electrode surface, and an electrochemical surface area of 3.784 cm^2^ and an average catalytic rate constant of 195 mol^−1^ L s^−1^. The sensor demonstrated a linear response from a cholesterol concentration of 0.04 to 600 mg dL^−1^ at a sensitivity of 226.30 μA mM^−1^ cm^−2^. The LOD and the LOQ were determined to be 0.00204 and 0.0062 mg dL^−1^, respectively. Measurements on the clinical samples showed promise in further clinical testing.^72–74^

A composite combined glucose and cholesterol biosensor of Fe_3_O_4_ dispersed on silica-coated (SiO_2_) CVD-grown MWNTs was reported. Non-enzymatic cholesterol sensing was performed by applying the material onto a GCE with Nafion binding and protective agent. The active material functioned as an electron transfer mediator resulting in an increased current response in cholesterol oxidation/reduction. The presence of Fe^3+^ and Fe^2+^ ions enabled the detection of H_2_O_2_ produced during cholesterol oxidation. The composite biosensor exhibited a linear detection range between 10 μM and 4 mM, an LOD of 5 μM, and a rapid response time of 5 seconds. Furthermore, stability tests conducted over a 2 month-period demonstrated a stable response, and a 90% response retention after 15 consecutive measurements for both glucose and cholesterol.^75^

**Fig. 3:**
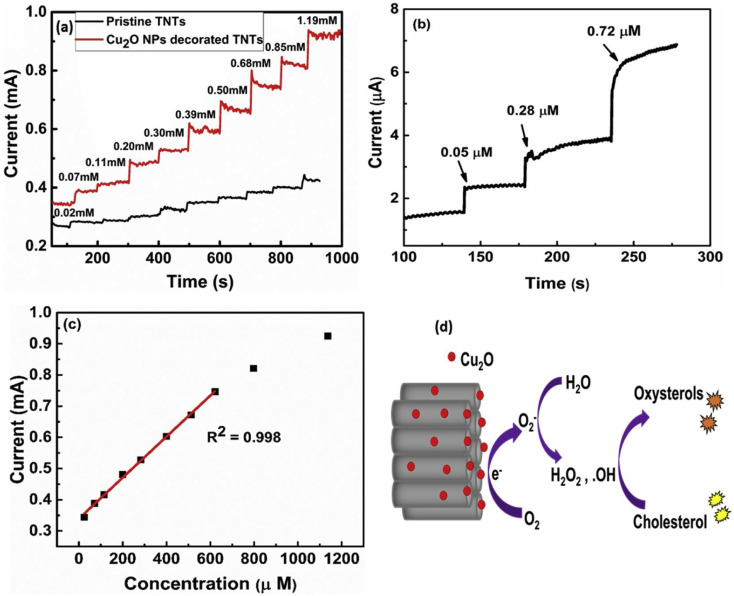
Amperometric response of the pristine TNTs and Cu_2_O decorated TNTs at −0.46 V in 0.1 M PBS at pH 7.0 upon successive addition of cholesterol in the (a) high, and (b) low concentration ranges. (c) The corresponding linear calibration curve at high cholesterol concentration, and (d) schematic of electrochemical oxidation of cholesterol at the surface of the Cu_2_O NPs decorated TNTs electrode, reprinted with permission from ref. 77. Copyright 2020 Elsevier and Copyright Clearance Center (LN: 5833130132597).

Khaliq *et al.* developed an electrochemical cholesterol sensor based on a hybrid nanostructure composed of Cu_2_O and TiO_2_ nanotubes. The latter was synthesized from a pretreated Ti foil through a two-step anodization procedure followed by annealing,^78^ while the subsequent Cu_2_O nanoparticle deposition was done by cluster beam deposition (CBD). The cholesterol sensing performance was verified by electroanalytical techniques (CV, EIS, CA *etc.*) in PBS/IPA, and the quantification based on amperometry as shown in Fig. 3a–c. A linear detection range (LDR) spanning from 24.4 to 622 μM was found with an LOD of 0.05 μM along with a high sensitivity of 6034 μA mM^−1^ cm^−2^, and a fast response of 3 seconds. The copper's redox behavior was identified as the driving mechanism behind cholesterol sensing as illustrated in Fig. 3d. Cu_2_O first adsorbs oxygen (O_2_) and then generates O_2_^−^ ions, which subsequently react with water and forming hydroxyl radicals (OH˙). The latter initiates cholesterol auto-oxidation and ultimately leads to electron generation, which in turn provides an electrical signal connected to cholesterol concentration. Furthermore, a comprehensive clinical sample study was undertaken to assess the impact of interfering substances during the sensing process. The performance of the as-fabricated electrodes was comparable to that of certain commercial sensors, indicating the robustness and reliability of the developed system.^77^

**Scheme 3:**
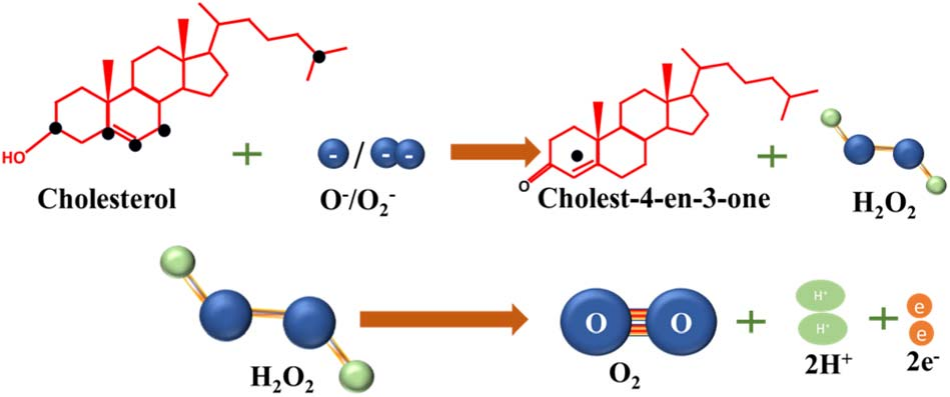
Reactions involved during non-enzymatic detection of cholesterol with the participation of oxygen ions.

Polycrystalline ZnO and Zn_2_In_2_O_5_ phases were deposited on a copper substrate and was utilized as a cholesterol biosensor. The electrode was fabricated onto a pre-treated copper substrate through the co-deposition of Zn and In from the aqueous solution of ZnCl_2_, KCl, InF_2_, and H_3_BO_3_, followed by hydrothermal oxidation. Cholesterol sensing tests were conducted in PBS buffer employing CV and DPV. During positive potential sweep, oxygen adsorbed on the electrode is converted into ionic species by extracting electrons from the electrode. These mobile oxygen ions (O_2_^−^/O^−^) participate in the reverse sweep, leading to the oxidation of cholesterol to cholestenone and the production of H_2_O_2_ as a byproduct. Subsequently, an electron is released due to H_2_O_2_ oxidation, and the transfer of this electron to the electrode reduces a metal ion back to its atomic form. The two-step process is outlined in Scheme 3. The electrode demonstrated a linear detection range for cholesterol concentrations between 0.5 mM and 9 mM, with a sensitivity of 81 μA mM^−1^ cm^−2^ and a fast response of 1 second.^79^

**Fig. 4:**
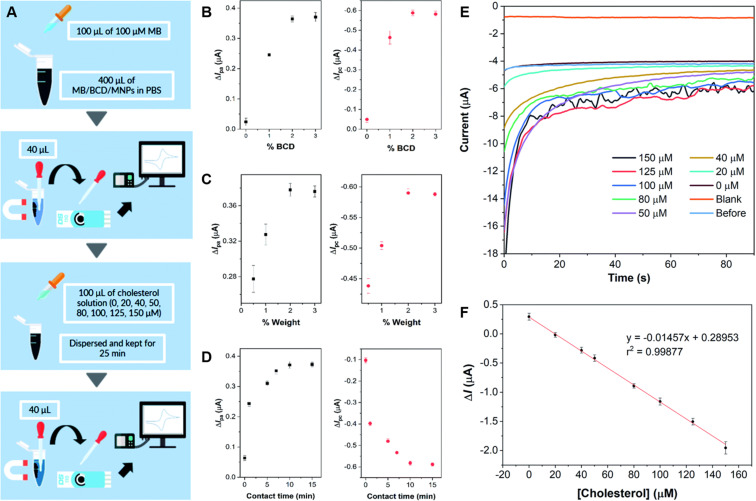
Schematics of the β-cyclodextrin/Fe_3_O_4_ nanocomposite cholesterol sensor fabrication process (A), plots of methylene blue current differences before and after the addition of 100 μM cholesterol in 50 mM PBS (pH 7.4) by cyclic voltammetry on screen-printed carbon electrode using nanocomposites synthesized with different BCD contents in BCD/MNPs (2% w/w) (B), different amounts of 3%-BCD/MNPs (C), and different contact times (D). Amperograms obtained for blank solution (50 mM PBS, pH 7.4), before and after cholesterol addition at concentrations between 0 and 150 μM at a potential of −0.43 V for 90 s under optimum conditions (E), and the corresponding calibration curve of the developed cholesterol sensor (F). Reprinted with permission from ref. 80. Copyright 2020 Royal Society of Chemistry (LN: 1507238-1).

Willyam *et al.* constructed a non-enzymatic cholesterol biosensor by combining magnetite (Fe_2_O_3_) nanoparticles (MNP) and β-cyclodextrin (CD). The composite was synthesized by the co-precipitation of Fe^2+^ and Fe^3+^ in BCD (MNP/BCD) under alkaline conditions, and the resulting precipitate was then separated with an external magnet. The active material was immobilized on SPCE, and cholesterol detection was achieved by adding methylene blue (MB) to the electrode, as sensing mechanism based on the competition of inclusion complex formation between cholesterol and β-cyclodextrin (BCD), and methylene blue (MB), respectively. The electrochemical behavior of the fabricated electrode was studied by CV, DPV, and CA, and the sensor showed a wide linear response range from 0 to 150 μM along with a low LOD of 8.11 μM, 4.77 μM, and 2.88 μM for the anodic and the cathodic CV peaks, and for utilizing amperometry, respectively. The schematic procedure of cholesterol sensing using the developed sensor, the corresponding current responses obtained from CV under various conditions and the amperometric response during cholesterol sensing are shown in Fig. 4. The sensor also displayed excellent repeatability and recovery in cholesterol determination in 50 mM PBS solution (pH = 7.4). It is worth noting, that further interference studies demonstrated that the presence of NaCl, CaCl_2_, glycine, glucose, and ascorbic acid at concentrations ten times higher than cholesterol did not significantly affect the accuracy of cholesterol measurements.^80^

A copper(ii) oxide/(CuO)/PANI/murexide (Mu) composite biosensor was fabricated on glassy carbon electrode *via* electrooxidation and electrodeposition. The electrochemical performance of the sensor was determined by CV, EIS, and linear sweep voltammetry (LSV), and showed high sensitivity and high stability across a broad concentration range from 0.5 to 1 mM. The developed bioanalytical system was applied to determine cholesterol concentration in milk with high recovery rates and high selectivity, demonstrating its potential as a valuable tool in real-life cholesterol detection.^14^

**Fig. 5:**
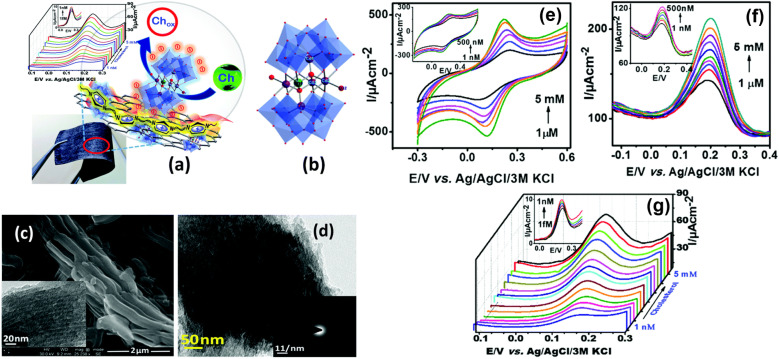
Schematic representation of cholesterol detection with the PVIM–Co_5_POM/MNC composite on a flexible paper electrode (a). Single crystal X-ray structure of Na_12_[WCo_3_(H_2_O)_2_(CoW_9_O_34_)_2_]; Na and H atoms are omitted for clarity (b). FE-SEM image of the MNCs (inset: TEM image of the MNC) (c), and TEM image of the PVIM–Co_5_POM/MNC composite (inset: SAED pattern of the same structure) (d). CV (e) and DPV (f) of the PVIM–Co_5_POM/MNC-600 modified graphite electrode at various concentrations of blood serum in 0.1 M phosphate buffer (pH 7.4) and 1 mM K_4_[Fe(CN)_6_] electrolyte in the presence of 500 μM cholesterol. DPV of the PVIM–Co_5_POM/MNC-600 modified flexible paper electrode at various concentrations of cholesterol in 0.1 M NaOH and 1 mM K_4_[Fe(CN)_6_] electrolyte at a scan rate of 10 mV s^−1^; CE: Pt wire, RE: Ag/AgCl/3 M KCl (g). Reprinted with permission from ref. 81. Copyright Royal Society of Chemistry (LN: 1507238-1).

A hybrid material comprising poly(ionic liquid) (PVIM) and cobalt polyoxometalate (Co_5_POM) anchored on carbonaceous materials (MNCs) was utilized as electrochemical cholesterol sensor, and demonstrated an unprecedentedly low LOD of 1 fM (1 × 10^−15^ M). The schematics of cholesterol detection, the structural details and some important electrochemical results are shown in Fig. 5. It showed a rapid response time of 5 seconds along with a wide dynamic detection range between 1 fM and 5 mM. The latter consists of two linear regions, one ranging from 1 fM to 200 nM and another spanning from 0.5 μM to 5 mM with a sensitivity of 210 μA μM^−1^ cm^−2^ and 64 μA μM^−1^ cm^−2^, respectively. Importantly, the impressive sensor performance remained unaffected in the presence of interfering substances. To validate its practical utility, the sensor was tested in a human blood serum sample at a physiological pH within a concentration range relevant to human biology. Furthermore, a flexible version of this sensor was also developed by coating PVIM–Co_5_POM/MNC onto a filter paper retaining the original high sensitivity and broad detection range, making it a versatile tool for a wide range of applications.^81^

Rabbani *et al.* deposited mesoporous NiCo_2_S_4_ nanoflakes onto nickel foam, and the fabricated electrode demonstrated a high sensitivity of 8623.6 μA mM^−1^ cm^−2^ across a broad linear range from 0.01 to 0.25 mM along with a considerable low LOD 0.01 μM. The NiCo_2_S_4_ structure also possesses robust thermal stability and consistent performance over the extended period of 8 weeks. Furthermore, the sensor exhibited high recovery in measurements on real-life blood samples.^82^

## Challenges and future perspectives

In the pursuit of direct cholesterol sensing through electrooxidation, researchers have to be aware of the oxidation potential of cholesterol, which is typically ≈0.4 V (*vs.* Ag/AgCl/sat'd KCl). It is crucial to take any potential variations due to the choice of reference electrodes and the specific oxidation sites within cholesterol into account. Furthermore, in order to have a mechanistic understanding of our system the redox properties of any functional moieties have to be known. It needs be ensured that these moieties are capable of either directly oxidizing cholesterol or participating in further redox reactions. Depending on the actual sensing layer, this could be reaction with byproducts H_2_O_2_, O_2_, *etc.*, or with intermediates such as oxygen, hydroxide ions, and radicals. Moreover, these moieties should serve as effective current capturers or sensors throughout the redox reaction process. Conducting *in situ* and *operando* analysis on intermediates and products during cholesterol oxidation, along with the investigation on elemental interactions between the electrode components and cholesterol, would significantly advance the field.

Cost-effective, stable, durable, and reproducible metal oxide-based electrodes with high sensitivity can compete with existing enzyme-assisted sensor systems. Tailoring the characteristics of metal-based systems to possess inherent cholesterol-oxidizing capabilities eliminates the need for additional activating agents, and ensures the development of an easily monitorable current signals at the electrode. Additional materials can also be incorporated into the sensor chips either as filters to eliminate interfering components or as supporting reagents to achieve minimal or zero input current. Commercially viable sensor chips utilizing highly active metal(oxide)-based working electrodes with cost-effective counter and reference electrodes on suitable substrate would give further impetus to the continuously developing field of electrochemical cholesterol sensing.

## Summary

Non-enzymatic electrochemical cholesterol sensing techniques offer numerous advantages over its enzymatic counterparts, such as being enzyme-free, having a straightforward operating principle, maintaining high stability in various environments, and offering a wide array of synthesis strategies for electrode development. Among the materials explored for non-enzymatic sensors, oxide-based systems are particularly noteworthy due to their sensitivity, stability, and reproducibility. A wide range of material families were already studied, primarily based on the oxides of Mn, Fe, Ni, Cu, Zn, Mo, Ti, In, and Si. While many research focused on lab-scale experiments, a few studies already incorporated clinical sample analysis as well. The advancement of chip-based non-enzymatic metal oxide-based systems for clinical applications necessitates extensive research into mechanistic aspects of material and electrode development strategies, cholesterol sensing methodologies, and protocols for testing physiological samples. This review aims to provide insights to researchers in academia and industry, with the hope that commercially available cholesterol sensor system will be realized soon.

## Abbreviations

PANI: Polyaniline
PPy: Polypyrrole
MWCNT: Multiwalled carbon nanotubes
rGO: Reduced graphene oxide
SPCE: Screen-printed carbon electrode
β-CD: β-Cyclodextrin
ChOx: Cholesterol oxidase
ChE: Cholesterol esterase
HRP: Horseradish peroxidase
PBS: Phosphate buffer saline
CV: Cyclic voltammetry
CA: Chronoamperometry
LSV: Linear sweep voltammetry
EIS: Electrochemical impedance spectroscopy
DPV: Differential pulse voltammetry
LOD: Limit of detection
PGE: Pencil graphite electrode
PVDF: Polyvinylidene chloride (PVDF)
NMP: *N*-Methyl-2-pyrrolidene
SCE: Saturated calomel electrode
LOQ: Limit of quantification
NR: Nanorods
NP: Nanoparticles
*R*_ct_
: Charge transfer resistance
MO: Methyl orange
GCE: Glassy carbon electrode
DA: Dopamine
PMO: Poly methyl orange
CNF: Carbon nanofiber
CVD: Chemical vapour deposition
ACF: Activated carbon fibre
PVC: Poly vinyl chloride
TEOS: Tetraethoxysilane
TNT: TiO_2_ nanotubes
CBD: Cluster beam deposition
MNP: Magnetic nanoparticles
Mu: Murexide (Mu)

## Data availability

No primary research results, software or code have been included and no new data were generated or analysed as part of this review.

## Author contributions

The manuscript was written through contributions of all authors. All authors have given approval to the final version of the manuscript.

## Conflicts of interest

The authors declare no competing financial interest.

## Appendix: vocabulary

Cholesterol, a compound present in the foods you consume that serves vital functions in human body. Enzymes, biological catalyst accelerate chemical reactions without being consumed or permanently altered in the process. Biosensors is a specialized sensor that utilizes biological molecules or components to detect and measure specific biological or chemical substances, often used for applications in healthcare, environmental monitoring, and biotechnology. Analyte, a specific substance or chemical compound that a sensor or analytical instrument is designed to detect and measure its concentration within a given sample or environment. Electrode, in sensing is a specialized conductor used to detect, measure, or record electrical signals or changes in electrical properties resulting from interactions with the surrounding environment, which is essential for various sensing applications such as electrochemical, pH, or biosensors. The limit of detection (LOD) is the lowest concentration or amount of a substance that can be reliably detected, but not necessarily quantified with a particular analytical method or sensor. The limit of quantification (LOQ) is the lowest concentration or amount of a substance that can be accurately and precisely measured and quantified with a particular analytical method or instrument. According to IUPAC, LOD and LOQ is 3 and 10 times higher than the standard deviation of the blank measurement (background noise), respectively. Sensitivity, in the context of sensing refers to the ability of a sensor to reliably detect even small changes in the quantity or concentration of an analyte in a sample. It is, by definition, the slope of the calibration curve.
